# Dendritic cells in lung immunopathology

**DOI:** 10.1007/s00281-016-0571-3

**Published:** 2016-06-02

**Authors:** Peter C. Cook, Andrew S. MacDonald

**Affiliations:** Manchester Collaborative Centre for Inflammation Research, Core Technology Facility, The University of Manchester, 46 Grafton Street, M13 9NT Manchester, UK

**Keywords:** Dendritic cells, Lung, Th2, Th17, Allergies, Asthma

## Abstract

Dendritic cells (DCs) lie at the heart of the innate immune system, specialised at recognising danger signals in many forms including foreign material, infection or tissue damage and initiating powerful adaptive immune and inflammatory responses. In barrier sites such as the lung, the instrumental role that DCs play at the interface between the environment and the host places them in a pivotal position in determining the severity of inflammatory disease. The past few years has seen a significant increase in our fundamental understanding of the subsets of DCs involved in pulmonary immunity, as well as the mechanisms by which they are activated and which they may use to coordinate downstream inflammation and pathology. In this review, we will summarise current understanding of the multi-faceted role that DCs play in the induction, maintenance and regulation of lung immunopathology, with an emphasis on allergic pulmonary disease.

Dendritic cells (DCs) are critical innate immune cells at barrier sites, including the lung, playing a decisive role in initiation of adaptive immune responses against foreign material, infection, commensals or tissue damage [1]. Of all antigen presenting cell (APC) types in the body, DCs are particularly adept at recognising such dangers, migrating from the periphery to the lymphatics, and activating naïve CD4^+^ and CD8^+^ T cells [2]. While the discussion as to what truly distinguishes DCs from other mononuclear phagocytes continues in some circles, most researchers would agree that functional rather than phenotypic definition of DCs is the key. In this review, in line with the general consensus [1], we define as ‘DCs’ any mononuclear phagocyte that has the ability to take up antigen (Ag), process it for presentation on MHC-I or II, migrate to the nearest draining lymph node (dLN) and efficiently and effectively activate and polarise naïve T cells. Their crucial role in this regard is clearly illustrated by the fact that people with genetic defects resulting in a lack of DCs, or mice wherein DCs are experimentally depleted, suffer from severely impaired adaptive T and B cell responses and increased susceptibility to a wide range of infections [3, 4]. This role as the preeminent cell type in the body for activation and modulation of adaptive responses, and in determining the balance of effector cell vs. regulatory responses, is central to understanding the normal progression of protective immunity in most situations. As clonal expansion of adaptive immunity can directly amplify or regulate immunopathology, consideration of this key role of DCs in maintaining health in the lung is key.

## The allergy epidemic and allergic pulmonary disease

As a mucosal barrier site, the lung is continually exposed to a wide variety of challenges that can trigger immune responses that, when inappropriate or unbalanced, can cause immunopathology. Such responses underpin respiratory disease and are a significant cause of illness and mortality worldwide, with allergic responses leading to asthmatic disease having a dramatic impact on global health [5–7]. Immune mechanisms underlie allergic disease and the pathology associated with allergies, typified by a defined range of cell types and pathological responses. These include activation and/or recruitment of a variety of characteristic innate and adaptive immune cells, as well as induction of a range of distinctive physiological processes that together are sometimes termed as ‘type 2 inflammation’ [8].

In the lung, type 2 inflammatory mechanisms can result in allergic asthma, currently thought to be the predominant cause of asthma in children, and in approximately 50 % of adults [9]. Acute symptoms of asthma include wheezing, coughing and shortness of breath, characterised as airway hyperreactivity (AHR), that can be life threatening. Over time, chronic pulmonary type 2 inflammation can lead to damaging fibrotic pathology and leave individuals at increased risk of secondary pulmonary infections or chronic obstructive pulmonary disease (COPD). Immunological hallmarks of allergic asthma include eosinophilia in patient bronchoalveolar lavage (BAL) fluid, sputum or bronchial biopsies, Th2 polarised CD4^+^ T cells secreting type 2 cytokines (including IL-4, IL-5 and IL-13), and IgE antibody. However, in recent years, as patient stratification approaches have become more refined, it has become clear that asthma is not a single disease and that individuals with asthma can be further classified into distinct groups or ‘endotypes’ [10]. In particular, not all asthmatics present ‘classical’ eosinophilic/type 2 disease, with some patients instead displaying disease that is more dominated by neutrophils and insensitive to steroid treatment [11, 12]. As type 2 (eosinophilic) and type 17 (neutrophilic) inflammation are thought to be governed by different cellular and molecular mechanisms [13], this may help explain the modest levels of success so far of therapies that have tried to target type 2 inflammatory mediators for all asthma patients. Implicit in these processes is the character of the immune and inflammatory response that an individual develops, which directly impacts the type and severity of resulting immunopathology.

Type 2 inflammation is a characteristic of allergic inflammation, but also of parasitic helminth (worm) infection [14–16]. The effector cell types and mechanisms at play in both allergic and helminth infection settings appear to be very similar, being dominated by eosinophilia, CD4^+^ Th2 cells, alternatively activated macrophages and GATA3^+^ type 2 innate lymphoid cells (ILC2s). However, it could be argued that, while the type 2 response against parasitic worms has likely evolved ‘appropriately’ to enable the host and parasite to coexist effectively, the response against allergens is likely an aberrant or ‘inappropriate’ overexuberant response to foreign material that is not inherently dangerous [17, 18]. Related to this, the type 2 response against worms has also likely evolved to heal tissue damage associated with migration of these often large multicellular organisms through the body, as rapidly and effectively as possible [19–21]. Further, when dysregulated, these very same healing mechanisms can lead to damaging fibrotic pathology [22].

Type 17 inflammation is more characteristic of fungal infection, and typified by neutrophilia and CD4^+^ Th17 cells with the ability to make a distinctive range of cytokines, including IL-17A (which we will refer to as IL-17) [23]. Remarkably, although there are numerous triggers of asthma that are abundant in the environment such as pollution, allergens such as house dust mite (HDM), animal dander, pollen and fungal spores, of the 0.5 million deaths attributed to asthma each year worldwide, 50 % may have fungal asthma (GAFFI, 2016). Intriguingly, fungi have the capacity to stimulate both type 2 and type 17 inflammation that may or may not confer immune protection [24–29]. As such, fungal allergens may be of particular interest in trying to understand the mechanisms that dictate the balance of type 2 and type 17 pulmonary disease.

## DC activation and induction of adaptive immunity in the lung

DCs reside throughout the lung tissue, underlying the epithelial layer, poised to encounter foreign material, infections or tissue damage. In this role, they are aided by their ability to actively sample the airways [30]. Although present at low numbers, DCs are highly sensitive to their environment, expressing a variety of pattern recognition receptors (PRRs) including toll-like receptors (TLRs), C-type lectin receptors (CLRs) and Nod-like receptors (NLRs). Through these receptors, they are able to sense a wide range of pathogen, microbe or damage-associated molecules, ranging from bacterial, viral, fungal and protozoal commensals and pathogens through to allergens, particles and pollutants. Additionally, the pulmonary environment presents some unique features relative to other barrier sites, such as specific surfactants and mucins, which can also influence DC activation and function. Importantly, the combination of PRRs engaged can tailor the developing immune response by directly influencing the activation and function of DCs.

### The influence of other innate cells on pulmonary DCs

As well as responding to these environmental stimuli directly through PRR engagement, DCs can also be indirectly activated by other innate cells in the local environment. The pulmonary epithelium is a complex tissue, consisting of a variety of epithelial cell (EC) types including ciliated ECs, goblet cells and Clara cells which vary in composition and distribution depending on their location within the lung [31, 32]. While each of these cell types can act in their own right as innate protection against barrier disruption, infection and damage, their interaction with DCs can also alter the developing adaptive immune response [31]. A range of EC mediators and cytokines, in particular IL-25, IL-33 and thymic stromal lymphopoietin (TSLP), is thought to be important for conditioning of DC activation and function and for Th2 induction [33]. In addition, EC-derived endogenous danger-associated molecular patterns (DAMPS) such as ATP [34] and uric acid [35] can also impact DC activation and function in the lung, being triggered in by allergens such as HDM, with the balance of these signals being crucial for mediating allergic inflammation. Though less is known about the role of EC-derived cytokines in the context of neutrophilic asthma, recent evidence confirmed that EC TLR4 expression can mediate eosinophil inflammation in the airways, yet is dispensable for neutrophil influx, which instead depends on haematopoietic TLR4 sources [36]. Furthermore, IL-17 can directly trigger ECs to induce recruitment of neutrophils via CXCL1 [37].

The identity of which specific EC types produce which combinations of mediators in type 2 and type 17 settings, and how this specificity may be selectively controlled, is only beginning to be defined at mucosal sites [38]. Related to this, while EC recognition of endotoxin (bacterial lipopolysaccharide) present in the lung via TLR4 promotes allergic responses [39], chronic exposure to LPS modulates EC responses and dampens DC ability to induce allergic inflammation against HDM [40]. This is a key point, as the lung contains a stable microbiome that is dysregulated during allergic inflammation [41–43]. Further, gnotobiotic mice display exacerbated allergic inflammation [44] and neonatal mice that are yet to establish a diverse microbiota mount enhanced allergic responses to HDM, with the microbiota inducing DC tolerance to allergens [45]. The relevance of this has been demonstrated that exposure to high bacterial environments such as farms in childhood reduces the risk of allergy and asthma [46].

Much less is known about the role of endothelial cells in these processes, though it is clear that angiogenic remodelling is a feature of asthma [47]. It seems likely that they will be heavily involved in settings where danger signals reaching the lung are blood-borne, influencing the migration of immune cells such as eosinophils to and from the lung tissue site [48].

ILCs comprise a diverse grouping of subsets, distinguished based on the transcription factors they express and the cytokines they produce [49]. Although some agreement now exists in terms of ILC genealogy and diversity, vigorous debate still surrounds discussion of their relevance in most settings. Present in very small numbers in the lung, ILC2s expand in response to allergic stimuli and EC production of cytokines such as TSLP and IL-33 [50] and TGFβ1 [51]. ILC2s can clearly produce large amounts of IL-13 in particular which, in addition to directly impacting pathology, may feed back to influence activation, cytokine/chemokine production and migration of DC subsets from the lung [52, 53]. In this context, the epidermal growth factor (EGF)-like cytokine amphiregulin (AREG), which can be produced in substantial quantities by ILC2s, has been linked to a variety of outcomes that could directly or indirectly influence immunopathology in the lung [54]. Compared to ILC2s, less is known about how ILC3s may influence development of neutrophilic airway inflammation through production of cytokines such as IL-17. A recent study found that obese mice spontaneously developed AHR with significant numbers of CCR6^+^IL-17^+^ ILC3s and identified ILC3s in the BAL fluid of asthma patients [55]. It remains to be determined what triggers ILC3 recruitment during asthma, as well as what impact they have on DC ability to promote allergic airway inflammation.

Other innate cell types that can influence developing immunopathology in the lung via their impact on DC activation and function include granulocytes such as eosinophils, basophils and mast cells. For example, eosinophils can impact DC activation and type 2 induction through their degranulation and secretion of granule components such as the alarmin eosinophil-derived neurotoxin or eosinophil peroxidase [56, 57]. Basophils in particular, though dispensable for Th2 induction *per se*, have been suggested to provide an important accessory cell role as sources of IL-4 in supporting chronic type 2 inflammation [58–61].

### DC coordination of innate and adaptive inflammation in the lung

**Fig. 1:**
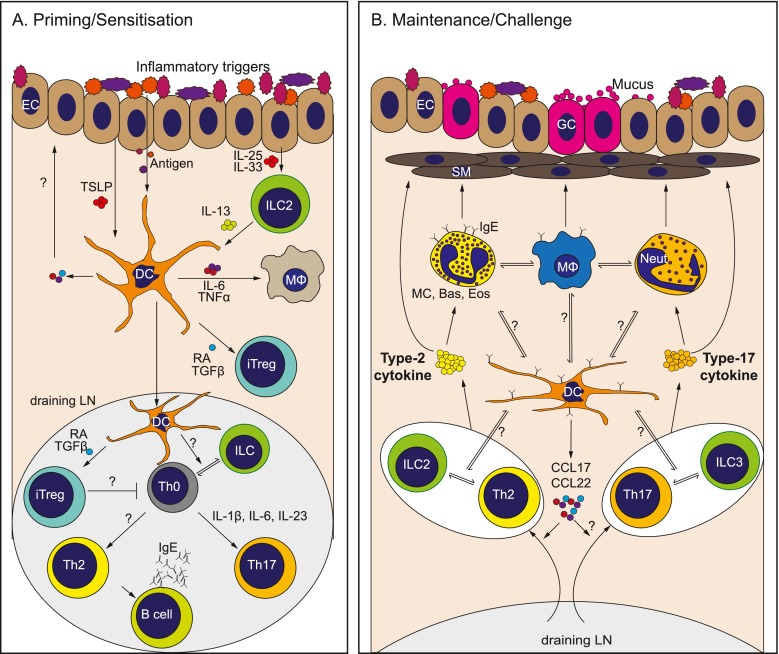
Key players in type 2/type 17 pulmonary inflammation and immunopathology. **a** The lung is continually exposed to a variety of signals that can trigger immune responses. In a healthy lung, exposure of epithelial cells (ECs) to danger signals triggers damage responses and the release of tissue factor cytokines such as TSLP IL-25 IL-33 and TGFβ [39, 50, 51, 173]. Simultaneously, antigens/allergens from the airspaces directly activate dendritic cells (DCs) located in the lung mucosa under the epithelial barrier [59, 86, 126]. The tissue factors released by the epithelium can not only influence the DCs directly but also indirectly via other innate cells such as innate lymphoid cells (ILCs) to alter DC activation or migration and promote allergic inflammation [52, 53]. Activated DCs can secrete a variety of proinflammatory mediators such as IL-6 and TNFα that may influence other innate cells in the lung such as intersitial macrophages (MΦs). Simultaneously, these DCs can also secrete regulatory cytokines such as TGF-β, promoting the generation of inducible regulatory T cells (iTregs) [152]. DCs then migrate to the draining lymph node (LN) via CCR7 to prime and polarise Th2, Th17 and iTreg cells, a process that may be assisted by cytokine production by accessory cells such as ILCs. The mechanism(s) by which this is achieved, and how these responses are balanced, is not yet clear. **b** During chronic exposure to inflammatory signals from antigens/allergens and/or innate cells, DCs release chemokines such as CCL17 and CCL22 to recruit effector Th2 and Th17 cells to the lungs, promoting the type 2/type 17 allergic inflammatory environment. It is unknown the extent to which DCs collaborate with other innate cells such as ILCs in the activation of recruited T cell populations, or innate effector cells such as granulocytes (MC; mast cells, Bas; basophils, Eos; eosinophils) and MΦs, to maintain chronic type 2 or type 17 inflammation in the lung. In type 2 settings, this process is also influenced by B cells releasing IgE that can bind to FcR-expressing innate cells. In contrast, type 17 responses promote neutrophil (Neut) infiltration, which may also impact activation and function of innate effector populations such as MΦs [174]. In both type 2 and type 17 settings, soluble mediators from innate and adaptive effectors can then mediate disease features such as goblet cell (GC) hyperplasia and smooth muscle (SM) contraction [63–65, 78]. Thus, DCs play a central role in promoting or regulating chronic pulmonary disease (1) through their central ability to initiate and direct primary responses and (2) through influencing effector cell recruitment and activation during ongoing inflammation. They achieve this through cross-talk with a diverse range of innate and adaptive effector populations, via production of soluble mediators and/or direct cell contact. Defining the specific DC subsets involved and the precise mechanisms they employ to communicate with these diverse effector cells, and how this changes over the course of chronic inflammatory disease and in different disease settings, will reveal new avenues for therapeutic intervention

Depending on the combination of the above factors - PRR engagement, the influence of other innate cells types and the unique lung environment - the outcome will be DC activation or ‘maturation’ to express a diverse array of surface molecules or mediators that can dramatically influence lung disease development (Fig. 1). The secretion of proinflammatory or antiinflammatory cytokines in response to local activating triggers is the initial, and often overlooked, innate mechanism by which DCs can exert influence over immunopathology in the lung in the very early stages of infection or tissue damage. Although present in very low numbers in the tissues, cytokine production per cell can be substantial [2]. In this way, DCs can rapidly produce high levels of proinflammatory cytokines such as IL-12, TNFα and IL-6 in response to PRR ligands, and in doing so, dramatically shape the character of the local inflammatory environment. For example, DC-derived IL-12 can act directly on NK cells to trigger their activation and IFNγ production in what could be deemed a ‘bystander’ fashion [62].

However, the main mechanism by which DCs impact immunopathology in any setting, including the lung, is through their potent ability to activate and influence adaptive arms of the immune system. Following their activation in the lung, DC migration to the mediastinal LNs enables them to interact with and activate naïve T cells that, through rapid clonal expansion and generation of effector cells that produce high levels of cytokine, and immunological memory, can have a major influence over developing and chronic immunopathology.

Effector and memory Th2 cells can exert a range of pathological outcomes in the lung, primarily through the impact of the cytokines they produce. These include granulocyte recruitment, goblet cell hyperplasia (leading to mucus overproduction) and bronchoconstriction [63–65]. Type 2 cytokines, and in particular IL-13, have been proposed to narrow airways in vitro and in vivo by triggering expression of the small GTPase RhoA, which drives muscle contraction [66]. However, other work involving cell-specific deletion of IL-4Rα on airway smooth muscle cells has proposed that muscle constriction in type 2 airway inflammation can occur independently of IL-13 [67]. In addition, in asthmatic airways, long-term inflammation results in a dysregulated wound healing response, which causes extensive collagen deposition and subepithelial fibrosis that involves cytokines such as IL-10 and IL-13 [68]. Further, as well as their direct roles in pathology, some of these Th2-driven outcomes feedback to affect ongoing DC function. For example, the mucin Muc5B has been shown to influence DC maturation in the context of tumour cells [69], though more work is needed to determine how goblet cell hyperplasia and mucins influence DCs to shape allergic inflammation in the lung.

As introduced above, it is increasingly clear that asthma is a broad spectrum of disease and some patients develop a more type 17-associated disease, with dominance of neutrophils rather than eosinophils [70], along with steroid resistance [71]. Several different cellular sources of IL-17 have been implicated in lung inflammation, including Th17 cells, γδ T cells, ILCs and even macrophages [72]. The situation is clearly more complex than originally thought, and the mechanisms that determine type 2/type 17 coexistence and balance during lung allergic inflammation are only beginning to be identified. The ability of core elements of type 2 inflammation such as IL-4 [73, 74] or ILC2s [75] to regulate IL-17 is well documented. In contrast, and somewhat counterintuitively, IL-17 may actually promote type 2 responses in some settings [76], including allergic lung inflammation [77]. This suggests a sequential situation where IL-17 may initially promote some aspects of type 2 inflammation, only to be downregulated by type 2 mechanisms once they have established. In addition, IL-17 may be able to directly trigger lung smooth muscle contraction during asthma [78]. More recently, evidence of products of alternatively activated macrophages such as the chitinase-like molecule Ym-1 promoting lung neutrophilia in a type 2 context highlights the complexity of allergic lung pathology [79]. It seems likely that the factors involved in determining the type 2/type 17 balance will be complex and context-dependent. Given that DCs are centrally involved in promotion or regulation of both type 2 and type 17 inflammation, it is likely they will play a decisive role in determining this balance.

### How and where do DCs influence pulmonary T cell activation?

DC migration from the lung to the LNs is a process thought to require upregulation of the chemokine receptor CCR7 and responsiveness to the chemokines CCL19 and 21, which direct DCs to the regions of the LNs where they can interact with and activate naïve T cells [80, 81]. Once this interaction is initiated, DCs provide naïve T cells with several important activating signals [2]. The first (‘signal 1’) requires presentation of antigenic peptide to CD4^+^ or CD8^+^ T cells in the context of MHC-II or MHC-I, respectively. At this stage, the affinity or avidity of MHC/Ag for a particular T cell receptor, and the concentration or dose of Ag, can alter the ‘strength’ of activating signal delivered to the responding T cell [82]. Recent development of techniques such as multiphoton microscopy has highlighted the importance of the strength or duration of DC/T cell interaction for naïve T cell activation and polarisation [83]. The second (‘signal 2’) provides costimulation and survival signals to the responding T cell, for example through CD80 or CD86 interaction with T cell CD28. DC provision of signal 1 with inadequate levels of signal 2 can lead to T cell anergy or hyporesponsiveness, rather than activation and clonal expansion, and so is a key step in T cell activation and generation of functional immunity. In addition to these fundamental DC/T cell interactions, further factors at the time of T cell activation termed ‘signal 3’ can influence the polarisation of the T cells [84]. Signal 3 generally refers to cytokines produced by DCs that directly influence the naïve CD4^+^ T cell polarisation process, such as IL-12 promotion of Th1 development through T-bet induction and stabilisation. In addition to providing survival and activation signals, some costimulatory molecules may also act to influence T cell polarisation or function, as has been proposed for CD70 during Th1 induction [85]. Thus, it is the combination of the type and level of expression of signals 1, 2 and 3, all ‘encoded’ in DCs by the nature of stimulus they have encountered in the lung, that is the major determining factor in their T cell activation and polarising ability in the LNs.

For type 2 immunity, this process likely involves the help of cytokines derived from accessory cells such as ILC2s or basophils, while cells such as ILC3s could conceivably assist DCs in Th17 induction, though this has yet to be shown. In addition, CD4^+^ T cells activated by DCs provide help via costimulation and cytokine production to B cells to dramatically impact their activation and antibody production. During allergic asthma, type 2 cytokines (particularly IL-4) induce high level IgE production by B cells that can then exert a systemic influence over granulocyte activation in the lung, for example through Fc receptor cross-linking and sensitisation of mast cells leading to heightened release of pathological mediators such as histamine. The cumulative result is clonal expansion of antibody producing B cells along with effector T cells that can then leave the LN and migrate back to the lung to exert their influence over the pathological processes at play. Indeed, it has been proposed that pulmonary DC expression of the IgE receptor FcεRI is required for their mediation of ongoing HDM responses [86] and promotion of mucous metaplasia [87].

Th2 cell migration from the LNs to the lung tissue is thought to predominantly involve their expression of the chemokine receptor CCR4, increasing responsiveness to the chemokines CCL17 and CCL22 [88, 89]. CCL17 and CCL22 can be produced by a range of innate cells within the lung, including DCs [90, 91], macrophages [92] and ECs [93], are induced by cytokines such as TSLP, IL-25, IL-33 and IL-13 [94–96] and are often found at elevated levels during either murine or human pulmonary allergic inflammation [86,91,97–99]. Importantly, although CCR4 is often considered to be particularly important for Th2 cell recruitment [53, 88], it can be expressed by a variety of other cell types [100], including Th17 cells [101] and ILCs [102], implying that Th17 and ILC recruitment could also involve CCL17 and CCL22. These chemokines may also feedback to influence activation of DCs and ECs, as both have been reported to express CCR4 [103, 104]. Given the variety of cell types that can express CCR4, CCL17 and CCL22, identification of the key producers and responders will be important to better understand migration of effector CD4^+^ T cells to defined regions of the lung tissue during distinct phases of allergic pulmonary inflammation.

While the consensus would be that the majority of adaptive priming by pulmonary DCs occurs in the secondary lymphatic organs [105], tertiary lymphoid structures termed inducible bronchus-associated lymphoid tissue (iBALT) can be found in the lung during disease and may provide additional locations for DC/T cell interaction and activation [106]. In fact, it has been suggested that iBALT may even act as a site for naïve T cell priming, in a process that is maintained by and requires DCs [107, 108], as well as being a location for enhanced B cell activation. Given increasing awareness of the multifunctionality of other innate cell types such as ECs or ILCs present in the lung tissue, it may be expected that such cells would exert even more of an influence over the process of DC polarisation of naïve T cells in the iBALT than in the more remote LNs, in particular through provision of cytokines that may impact DC functionality, or alter polarisation of responding T cells directly.

It seems likely that ILCs may play an important accessory role as cytokine sources to enhance immune priming in LNs or iBALT, or influence effector or memory cell function in tissue sites. Further, as ILCs can express MHC [109–111], it remains possible they could act in some settings as APCs, though whether they have the capacity to effectively take up and process Ag, migrate to LNs and express sufficient costimulatory molecules to activate rather than regulate naïve T cells remains open to debate. Indeed, questions still remain about the relevance of ILCs in general in disease settings: how they are recruited, renewed or the location(s) in vivo where they may interact with and exert their influence over DCs. Determining these factors will be important to enable balanced judgement about their general importance, either directly or indirectly, in the development of immunopathology in the lung, particularly in chronic settings.

### DC subsets and induction of type 2 and type 17 inflammation

Although DCs have been shown to be both sufficient and necessary for Th2 induction in either helminth or allergic type 2 inflammation [59, 112, 113], the specific DC subset(s) required and the precise mechanisms they employ to promote type 2 inflammation and immunity, remain unclear [14,114–116]. However, some progress has been made on this front in recent years, as the details of DC ontogeny have been unravelled. DCs comprise a heterogeneous variety of cell types or ‘subsets’, which differ based on tissue location and/or expression of particular surface markers or RNA profiles. These subsets include conventional (or ‘classical’) DCs (cDCs), plasmacytoid DCs (pDCs) and—particularly during inflammation—non-conventional monocyte-derived DCs (moDCs, also sometimes called TNFα/iNOS producing DCs, or TIP DCs) [117]. In recent years, identification of defining transcription factors has enabled unambiguous classification cDC and pDC subsets in both mouse and man [118]. In the mouse, cDCs as defined by expression of the transcription factor Zbtb46 [119, 120] can be separated into two main subsets based on expression of the transcription factors BATF3 or IRF4, whereas pDCs express the transcription factor E2-2 [121]. *Batf3* expressing cDCs express CD8α^+^ in the spleen and CD103 or CD24 in the periphery, whereas IRF4^+^ cDCs express CD11b and are negative for CD8α. In man, cDC subsets exist expressing CD1c or CD141 in mucosal tissues that appear to be the equivalents of murine CD11b^+^ and CD8α^+^/CD103^+^ subsets respectively [122, 123], with similar expression patterns of transcription factors such as IRF4 in the lung [124].

In the healthy lung, the major populations of DCs to be found are present in the tissue rather than in the airspaces. CD103^+^ DCs are heavily associated with the pulmonary epithelium, while the location of CD11b^+^ DCs is predominantly in the underlying tissue [125, 126]. Migration studies have shown that, in the murine lung, CD11b^+^ DCs preferentially acquire and transport soluble Ag, whereas CD103^+^ DCs are more adept at dealing with particulate material [127]. From the limited ‘steady state’ human lung DC phenotyping data available, equivalent subsets can be identified, again predominantly in the tissues [122, 128].

Recently, IRF4-dependent CD11b^+^ cDCs have been associated with both Th17 [124, 129] and Th2 [130, 131] response induction and development. This diversity in CD11b^+^ cDC function likely relates to the heterogeneity that exists within this subset, and the fact that understanding of the transcriptional control of this diversity is still less developed than that of CD8α^+^/CD103^+^ cDCs or pDCs [132]. Although the precise IRF4-dependent cDC subset responsible for Th2 induction has yet to be unambiguously shown, CD11b^+^ cDCs that are reliant on the transcription factor Klf4, itself downstream of IRF4, have recently been implicated [133]. It is currently unclear how these CD11b^+^ IRF4- or Klf4-dependent cDCs relate to the CD301b^+^ DCs that have also been implicated in Th2 induction against parasitic worm infection and allergic responses in the skin [134] and type 17 inflammation to *Streptococcus* lung infection via production of IL-6 [135].

While it is becoming clear that CD11b^+^ cDCs may be the dominant cDC type involved in promotion of type 2 or type 17 inflammation, the role of CD8α^+^/CD103^+^ cDCs and pDCs in these settings is less well understood. Limited work in this area so far suggests that while CD8α^+^/CD103^+^ cDCs are particularly adept at promotion of Th1 responses and cross presentation to and activation of CD8^+^ T cells, they are dispensable for Th2 induction [136]. Similarly, while pDCs are characterised by their capacity to produce large amounts of type I IFN in response to viral infection and limited APC ability [137], they do not appear to be vital for Th2 induction against either allergens [138] or helminths [139]. Rather, it appears that both CD8α^+^/CD103^+^ cDCs and pDCs may in fact help to suppress or counter-regulate type 2 inflammation [136, 138, 140], though the precise mechanisms involved in this are currently unclear.

### DC subsets during inflammation

In both mouse and human, there is currently a huge disconnect in our understanding of the diversity and impact of DC subsets during any inflammatory setting, including in allergic pulmonary disease: most of our understanding in this area has been developed through study of lung tissue in the steady state, in the absence of overt inflammation. As more refined and higher resolution techniques such as mass cytometry [141], multiparameter flow cytometry and histocytometry [142] are increasingly applied to the lung, our understanding will expand to give vital insight into the diversity, location and activation state of DCs, their interaction with other key cell types and how this may change during disease.

What is likely, in both mouse and human, is that during lung inflammation more DCs can be found in the BAL and the proportion of moDCs present in both BAL and lung tissue increases. In murine models of eosinophilic asthma, CD11b^+^ DCs accumulate with effector T cells around the airways following allergen challenge [126]. Additionally, it is clear in such models that CD11b^+^ cDCs are superseded by CD11b^+^ moDCs as the predominant DC subset involved in Th2 induction with increasing allergen challenge [86]. Monocytes in general will likely play a much more dominant role in pulmonary inflammation and pathology than is currently appreciated, given increasing awareness of the impact of local and systemic inflammation on conditioning of monocytes early in their bone marrow development that can influence their resulting function in the periphery [143,144] and the multifunctionality of monocytes themselves [145].

As DCs play such a pivotal role in dictating the balance of adaptive immunity, determining in any given disease which subsets predominate, and the profile of activation that they present, may provide vital clues as to the main immunopathologic mechanisms involved. Importantly for asthma and pulmonary inflammation, whether and how CD11b^+^ cDCs or moDCs determine the balance between eosinophilic/type 2 and neutrophilic/type 17 inflammation remains to be determined and may be of particular relevance in situations such as fungal asthma.

Although the importance of DCs in priming and polarisation of naïve T cells in the lung draining LNs is clear, the role of DCs in recruitment, retention or reactivation of pulmonary effector or memory T cells during chronic inflammation is much less clear. In fact, the fundamentals of Th2 and Th17 cells are currently poorly understood in comparison to Th1 cells or CD8^+^ T cells, with little evidence that current concepts of T cell expansion, contraction and memory development/longevity are relevant in the context of Th2 or Th17 cells. However, recent work using HDM-specific MHC-II tetramers has suggested that a single exposure to HDM generates a central memory population of CD4^+^ T cells that remain in the lung to mediate AHR [146]. Further, Th2 effector cells have been shown to respond to IL-33 by exacerbating eosinophilic inflammation without the need for APC interaction [147]. Given that pulmonary disease is often chronic in nature, additional factors to consider include the development of T cell hyperresponsiveness over time and whether DCs may play a decisive role in fine tuning of memory T cell function.

## Immunogenic vs. tolerogenic roles for DCs in lung inflammation

As well as their potent immunogenic ability, DCs can also play a central role in regulation of inflammation and immunopathology [148]. Such ‘tolerogenic’ DCs are thought to be able to employ a variety of direct and indirect regulatory mechanisms, including secretion of antiinflammatory cytokines, induction of anergy or hyporesponsiveness and promotion of peripheral or induced regulatory T cells (iTregs) [149, 150]. In the steady state, lung DCs appear less able to induce Tregs than macrophages [151]. However, after allergen challenge, CD103^+^ DCs become strongly tolerogenic, producing both retinoic acid (RA) and TGFβ to generate iTregs [152]. In patients with COPD, tolerogenic DCs that induce IL-10^+^ iTregs have been identified [153], and *Helicobacter pylori* modulation of allergic inflammation is dependent on DC production of IL-10, yet independent of Tregs [154]. Given the ability of DCs to coordinate T cell effector function, including induction of iTregs, this is likely to have a profound impact on immunopathology in the lung involving any kind of adaptive component.

Interesting parallels exist between the phenotype of Th2 promoting DCs and some tolerogenic DCs, in particular a general low level or ‘immature’ phenotype evident in either case [114, 115]. Indeed, in some settings, Th2-inducing DCs, through their promotion of type 2 responses that can counter-regulate Th1 inflammation, could be considered regulatory or tolerogenic. As yet, a side by side comparison of Th2 vs. tolerogenic DCs has yet to be carried out but would likely be of real value in distinguishing mechanisms that are truly regulatory from those that are Th2 promoting. Further, the definition of immunogenic vs. tolerogenic features of DCs may relate directly to developing concepts of ‘innate memory’, where ‘trained’ DCs could display enhanced pro-inflammatory capacity, whereas ‘tolerised’ DCs would instead be regulatory, with the outcome being reduced or increased susceptibility to some infections [155], along with more or less severe inflammatory pathology [156]. As in other tissue sites, the nature of the stimuli that DCs encounter in the lung likely determines training vs. tolerance, and so immunogenic vs. tolerogenic capacity.

### DC mechanisms of T cell polarisation

Despite advances in subset identification and the general association of CD8α^+^/CD103^+^ DCs with Th1 [157] and CD11b^+^ DCs with Th17 and Th2 polarisation [124, 129–131,133,158], the specific mechanisms employed by DCs to promote type 2 inflammation and immunity remain unclear and a key unanswered question in the field [14, 114, 115]. The paradigm established over the past decades is that DC production of IL-12 is central to their ability to promote Th1 polarisation [159], though there are some exceptions to this general rule, such as the costimulatory molecule CD70 compensating for Th1 induction in the absence of IL-12 in some settings [85]. In type 17 responses, DC production of IL-1α, IL-6 and IL-23 has been strongly linked to their Th17 inductive ability, with variable reliance on each of these mediators depending on context, and when comparing murine and human DCs [160–162]. In terms of DC induction of iTregs, one cytokine that has been particularly implicated is TGFβ, and subsets of DCs can express TGFβ-activating integrin αVβ8 to facilitate this process [163]. In this context, as TGFβ can also act in concert with cytokines such as IL-6 to promote Th17 induction [160, 161], the relative balance of each cytokine at the time of CD4^+^ T cell priming could result in either a regulatory (iTreg) or an inflammatory (type 17/neutrophilic) outcome. In murine models of HDM or OVA-induced airway inflammation, mice with CD11c-restricted deficiency in αvβ8 displayed reduced CD4^+^ T cell IL-17 production but intact γδ T cell IL-17, iTreg and Th2 responses [78]. Together, this suggests that the dominant role for DC expression of αVβ8 in pulmonary inflammation is promotion of Th17 cells, though its potential role in Th2 induction, and which specific pulmonary DC subset(s) express this TGFβ-activating integrin, currently remains unclear.

In contrast to Th1, Th17 and iTregs, no DC-derived cytokine has yet been convincingly identified that is central to their Th2 induction ability. Indeed, data so far indicate that DCs tend to only display a limited maturation phenotype in response to allergens or helminths, with low level and non-distinctive cytokine production or changes in mRNA profiles, making it difficult to identify candidate mechanisms for type 2 induction [14, 114, 115]. While DC production of IL-4 or IL-10 is not a fundamental requirement for Th2 induction [164–166], it has recently been suggested that DC expression of PDL2 or IL-33, under the control of the transcription factor IRF4, may be important for this process [130, 131].

Although a clear DC-derived signal 3 for Th2 induction remains elusive, it is becoming increasingly clear that epigenetic mechanisms of transcriptional regulation may also play a major role in control of DC activation and function during T cell activation and polarisation and in allergic airway inflammation. The deubiquitinase A20 has been shown to limit DC activation and restrain their induction of inflammatory responses [167], while the methyl-CpG-binding protein Mbd2 is required for DC promotion of type 2 responses against either helminths or allergens [168]. This epigenetic layer of control of immune cells is not restricted to DCs, with A20, Mbd2 or the demethylase Jmjd3 also playing a part in control of innate cells such as macrophages and mast cells [169, 170], as well as, in some cases, adaptive T and B cells [171, 172]. Thus, defining the processes under the control of epigenetic regulators such as A20 and Mbd2 is an exciting new frontier that is already leading to discovery of previously unappreciated core mechanisms that innate cells such as DCs used in promotion or regulation of pulmonary inflammation.

## Conclusions

Although lung type 2 inflammatory disease can be triggered by a wide range of stimuli, DCs are centrally involved in the initiation and direction of the adaptive immune response and immunopathology that ultimately determines the severity of chronic disease. A better understanding of the DC subsets involved, the mechanisms that they employ and the cell types that they interact with during promotion, maintenance or regulation of inflammation in the lung will lead to the future development of novel therapeutics to combat the damaging immunopathology associated with chronic pulmonary inflammatory disease (Fig. 1).
